# Correlation between patency and clinical improvement after lymphaticovenous anastomosis (LVA) in breast cancer-related lymphedema: 12-month follow-up

**DOI:** 10.1007/s10549-019-05450-2

**Published:** 2019-09-21

**Authors:** Joost A. G. N. Wolfs, Luuke G. E. H. de Joode, René R. W. J. van der Hulst, Shan S. Qiu

**Affiliations:** Department of Plastic, Reconstructive and Hand Surgery, Maastricht University Medical Center, P. Debyelaan 25, 6229 HX Maastricht, The Netherlands

**Keywords:** LVA, Lymphaticovenous anastomosis, Breast cancer-related lymphedema, Patency, Quality of life

## Abstract

**Purpose:**

Breast cancer-related lymphedema (BCRL) is caused by an interruption of the lymphatic system after breast cancer treatment. Lymphaticovenous anastomosis (LVA), by which one or more patent lymphatic collecting vessels are connected to subcutaneous veins, shows promising results. Postoperatively, the patency of these anastomosis can be evaluated; however, little is known concerning the long-term patency after LVA in patients with BCRL. The aim of this study was to analyse the long-term patency, quality of life (QoL) and arm circumference after LVA, and to explore differences between patent and non-patent anastomosis and its correlation with clinical improvement.

**Methods:**

Twenty-five patients underwent indocyanine green (ICG) lymphography, lymph ICF-questionnaire, and arm circumference measurement preoperatively and 12 months after the LVA procedure.

**Results:**

Seventy-six percent of the patients showed at least one patent anastomosis after 12 months. Quality of life according to the Lymph-ICF increased significantly (*p* < 0.000); however, arm circumference showed no significant decrease. Sixty-five percent discontinued wearing compression stockings. The patent anastomosis group, compared with the non-patent anastomosis group showed, without significance, more improvement in QoL, arm circumference, and discontinuation of compression stockings, as well as a lower rate of infections both pre- and postoperatively, a shorter duration of lymphedema preoperatively, and a higher rate of early lymphedema and ICG stage.

**Conclusions:**

LVA showed an acceptable patency and positive correlation between a patent anastomosis and clinical improvement after 12 months. Further research with a larger study population is required to determine whether outcomes or patient characteristics significantly correlate with a patent anastomosis after LVA operation.

**Electronic supplementary material:**

The online version of this article (10.1007/s10549-019-05450-2) contains supplementary material, which is available to authorized users.

## Introduction

Secondary lymphedema in the upper extremity is caused by an interruption of the lymphatic system following cancer, trauma, injury, or infection. Breast cancer, the most frequent cancer among women, affects over 2 million women each year [1, 2]. Breast cancer treatment involving lymph node dissection or radiotherapy is the most common cause in developing upper extremity lymphedema, also known as breast cancer-related lymphedema (BCRL) [3, 4]. As a result, symptoms may negatively affect their quality of life (QoL) such as a swollen limb, pain, heaviness, and erysipelas [5–7]. Peripheral lymphedema can be classified clinically into severity stages according to the International Society of Lymphology (ISL) [8]. With the increasing breast cancer survival rate and the profound impact on QoL, lymphedema treatment is in high demand [5, 6, 9, 10].

Lymphedema treatment options aim to decrease symptoms and prevent the progression of chronic lymphedema. Complex decongestive therapy has an important role in alleviating symptoms of lymphedema [11–14]. However, as this standard treatment might be needed lifelong, super-microsurgical techniques have been introduced [15–18]. Koshima et al. performed the lymphaticovenous anastomosis (LVA) by which one or more patent lymphatic collecting vessels are connected to subcutaneous veins, thus restoring the physiological lymphatic drainage [19–22]. Using indocyanine green (ICG), a fluorescent marker, and a near-infrared camera, the lymphatic system can be visualised and dermal backflow patterns can be described by the so-called ICG stage [23]. These stages ranging from 0 to V describe ICG lymphography pattern changes from a normal linear pattern to an abnormal dermal backflow pattern in severe lymphedema (stage IV and V). After the lymphatic collecting vessels have been mapped, an artificial anastomosis can be made using the microscope [24–26]. Postoperatively, the patency of the anastomosis can be evaluated using the same ICG imaging technique. However, little is known concerning the long-term patency rate after LVA in patients with BCRL. Only few studies addressed the patency of LVA in upper extremity lymphedema [27, 28], and most of them examined lower extremity lymphedema in small study populations [27, 29–31]. Moreover, only few studies discussed the characteristics influencing the patency of LVA and concluded that more anastomosis per patient result in a higher chance of at least one patent anastomosis [28].

The aim of the current study was to analyse the long-term patency rate, QoL according to the Lymph-ICF, arm circumference, and compression stockings discontinuation after LVA, as well as exploring the differences between patients presenting patent or non-patent anastomosis and correlations with clinical improvement.

## Patients and methods

This study included consecutive patients who underwent a lymphaticovenous anastomosis procedure for breast cancer-related lymphedema in Maastricht University Medical Centre between January 2016 and May 2018. Approval of the IRB was obtained (METC 2018-0869). The inclusion criteria were: adult female patients, breast cancer-related lymphedema, viable lymphatic collecting vessels seen with a near-infrared ICG camera, and a minimum of 12-month follow-up period after LVA surgery. Patients with incomplete follow-up were excluded. Demographic data were documented, assembled retrospectively and analysed. Preoperatively and 12 months postoperatively, all patients underwent ICG lymphography, filled in the Lymph ICF-questionnaire for quality of life recording, and their arm circumference was measured.

### Surgical technique

The surgical LVA procedure as described by Koshima et al. was performed under local anaesthesia (bupivacaine hydrochloride 5 mg/ml with adrenaline 5 µg/ml) [19]. In brief, the lymphatic collecting vessels were located prior to the operation with the above-mentioned ICG lymphography. During the operation a small incision was made. Next, using a microscope, one or more anastomosis were performed between an adequate subcutaneous vein and a lymphatic collecting vessel using Ethilon 11-0. Finally, the patency of the anastomosis was checked intraoperatively and the skin was closed. In this study, patients were not allowed to wear compression stockings or receive manual lymphatic drainage (MLD) in the first month postoperatively in order to avoid harming the anastomosis. After that period, they could choose, in consultation with the plastic surgeon, whether to continue wearing the compression stockings or not, depending on the presence of subjective complaints and the presence of swelling in the arm. The plastic surgeon and skin therapist assessed if the MLD could be continued, reduced, or stopped.

### Outcomes

The patency of the anastomosis and QoL 12 months after LVA were the primary outcomes. The secondary outcomes were arm volume changes and the discontinuation of compression stockings. All variables were obtained both preoperatively (except the patency of anastomosis) and 12 months postoperatively.

### Patency measurement

Lymphatic system mapping and anastomosis examination was performed using a near-infrared camera (Fluobeam^®^; Fluoptics, Grenoble France) preoperatively and 12 months after LVA surgery. ICG 0.5%, 0.05 ml (PULSION^®^ 25 mg for solution, PULSION Medical Systems SE, Feldkirchen, Germany) was injected intracutaneously in the second and fourth webspace of the affected arm. Preoperatively, after 20–30 min, viable lymph vessels for LVA were located. Postoperatively, the patency of the lymphaticovenous anastomosis was assessed immediately after injection with the NIRF camera located right above the scar on the skin to identify the anastomosis. A short massage at the level where the lymphatic vessel is, assists the spreading of ICG through the anastomosis for better appreciation of the patency [19, 25, 26, 32]. Postoperatively, the patency was scored as follows: patent, non-patent, or not seen. The assessment of the score was done by two independent researchers. Only a clear flush of ICG through the anastomosis was scored as patent.

### Quality of life

Each patient completed the Dutch version of the Lymphedema International Classification of Functioning (LYMPH-ICF) questionnaire resulting in a VAS score from 0 to 100 to examine their quality of life preoperatively and 12 months after LVA surgery. The lower the score, the better the quality of life. An increase of more than nine or decrease of more than eleven points on this VAS score was considered statistically significant [33].

### UEL index

The arm volume changes in terms of circumference were measured according to the Upper Extremity Lymphedema Index (UEL index) [34]. After circumference measurement at standardised landmarks on the arm (olecranon, 5 cm above and below olecranon, wrist, dorsum of the hand), the UEL index was calculated from these circumference points and BMI [35]. Patients had to remove their compression stockings 24 h prior to the follow-up moment in order to achieve a reliable measurement.

### Statistical analysis

The patency rate and the discontinuation of compression stockings were indicated by percentages. For the QoL and the arm volume changes, a paired samples *T* test was used to examine effects pre- and postoperatively. Differences in outcome and characteristics between patent and non-patent anastomosis were only investigated exploratively by univariate analysis for the effect on patency of QoL, UEL index, age, BMI, and onset of lymphedema (ordinal variables), and a Fisher’s Exact Test for the effect of ICG stage, ISL stage, diabetes mellitus (DM), pre- and postoperative complications, and postoperative infection (categorical variables).

## Results

### Patients characteristics

Twenty-five female patients with a total of 47 anastomosis, and a mean of 1.9 anastomosis per patient, were included in the current study. Mean follow-up time was 14.8 months. In 3 patients, a postoperative wound infection occurred, which was successfully treated with antibiotics. The patients’ demographic characteristics are plotted in Table 1.

**Table 1:** Patient characteristics

Patient characteristics	Outcome
Number of patients	25
Sex	25 female, 0 male
Age (years)	58.4 ± 8.7
BMI (kg/m^2^)	25.8 ± 3.7
Smoking (%)	0
Diabetes mellitus (%)	DM1^a^ = 0, DM2^b^ = 8
ISL stage (%)	I = 8, IIA = 64, IIB = 28
ICG stage (%)	II = 28, III = 52, IV = 20
Affected arm (R/L) (%)	56 (R), 44 (L)
Years from lymphedema onset	6.2 ± 5.1 years
Preoperative infections (%)	32
Mean follow-up (months)	14.8 ± 5.7
Breast cancer treatment	
Radiotherapy (%)	84
Chemotherapy (%)	92
Lymph node dissection (%)	100
Mean number of lymph nodes dissected	15.1 ± 2.9
Mean number of positive lymph nodes	3.9 ± 3.8
Lymphaticovenous anastomosis	
Mean operating time (min)	97.2
Mean number of anastomosis	1.9

*DM1* diabetes mellitus type 1, *DM2* diabetes mellitus type 2, *ISL* International Society of Lymphology, *ICG* indocyanine green, *R* right, *L* left

### Patency

**Fig. 1:**
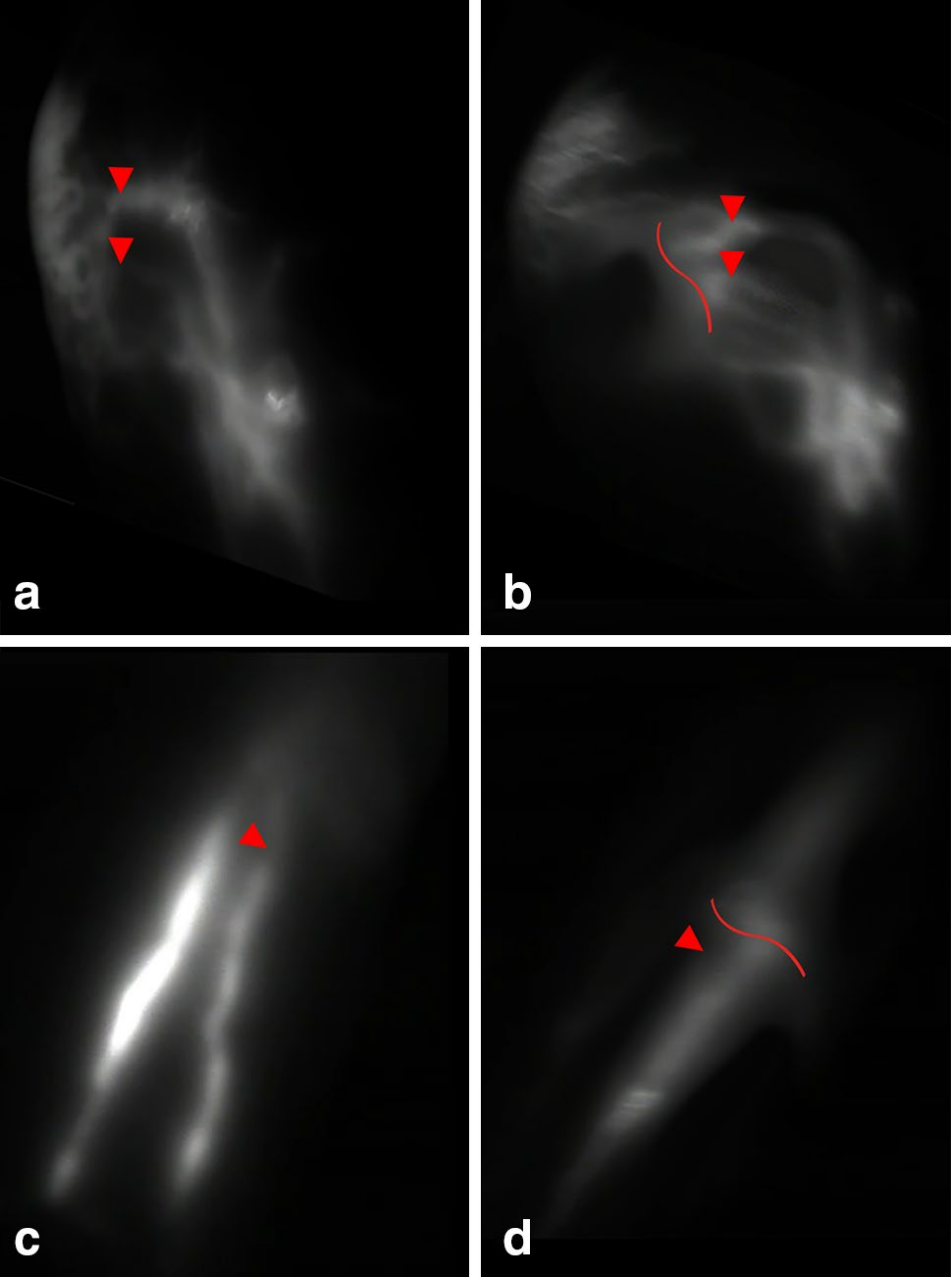
ICG lymphography with NIRF camera in 2 patients. Lymph vessels used for anastomosis are indicated by arrows, and location of the scar on the skin is indicated by curved lines. **a** Viable lymph vessels preoperatively and **b** non-patent anastomosis postoperatively (ICG flow stops at anastomosis site). **c** Viable lymph vessels preoperatively and **d** a patent anastomosis postoperatively

Postoperative examination with ICG lymphography demonstrated 19 out of 25 patients (76%) to have at least one patent anastomosis after 12 months. Figure 1 shows viable lymph vessels preoperatively and patent anastomosis postoperatively. In 2 patients, the anastomosis was not clearly seen and in 4 patients the anastomosis was non-patent. In total, 34 of 47 (72.3%) anastomosis were considered patent. An additional video of a patent anastomosis by ICG lymphography is given in Online Resource 1.

### Quality of life

The Lymph-ICF questionnaire showed a significant improvement on hand function (*p* = 0.001), mental function (*p* = 0.002), and mobility (*p* = 0.006) 12 months after LVA surgery. The household and social domain did not differ significantly (*p* = 0.275 and *p* = 0.222, respectively). As a result, the difference in total score pre- and postoperatively also showed statistical significant improvement, 47.5 and 31.5, respectively (*p* < 0.000).

### UEL index

The volume difference in terms of UEL index between the affected and unaffected arm was 16.2 preoperatively and 15.8 postoperatively, respectively. The difference, however, did not reach statistical significance (*p* = 0.822).

### Conservative treatment

Frequencies of conservative treatment pre- and postoperatively are shown in Table 2. Preoperatively, 80% of the included patients wore compression stockings. Twelve months after LVA surgery, 65% of these patients completely stopped wearing the compression stockings and 10% wore them to a lesser degree.

**Table 2:** Frequency of conservative treatment pre- and postoperatively

Conservative treatment	Preoperative (*n* = 25)	Postoperative (*n* = 25)
Compression stockings		
Yes	20	7
Daily	16	4
Not daily	1	2
Unknown	3	1
No	5	18
Manual lymphatic drainage		
Yes	21	17
> 1×/week	14	4
1×/1–4 weeks	4	11
< 1×/4 weeks	1	2
Unknown	2	0
No	3	7
Unknown	1	1

Eighty-four percent of the patients were treated with MLD by a skin therapist preoperatively, with the frequency ranging from 3 times a week to once in every 6 weeks. After 12 months, 38% continued MLD in the same frequency, and 38% to a lesser degree. Twenty-four percent of the patients completely stopped MLD. Wearing of compression stockings and MLD frequency were never intensified. As a result, the mean frequency of MLD pre- and postoperatively was once per week and once in 2 weeks, respectively.

### Patent vs. non-patent anastomosis

Differences in patients’ outcomes and characteristics between the patent and non-patent anastomosis groups are shown in Tables 3 and 4, respectively. Since none of the patients smoked and all diabetic patients showed a patent anastomosis, these variables were not analysed. Neither the outcomes nor patient characteristics between the patent and non-patent group showed significance after statistical analysis.

**Table 3:** Differences in outcomes between patent and non-patent anastomosis groups

Outcome	Patent anastomosis *N* = 19 (76%)	Non-patent anastomosis *N* = 4 (16%)	*p* value
Mean QoL	17.7 ± 18.7	11.5 ± 10.9	0.591
Mean UEL index	− 2.1 ± 10.0	4.2 ± 9.8	0.273
Compression stockings discontinuation (%)	75.0	25.0	0.089

*QoL* quality of life, *UEL* upper extremity lymphedema

**Table 4:** Differences in patient characteristics between patent and non-patent anastomosis groups

Characteristics	Patent anastomosis *N* = 19 (76%)	Non-patent anastomosis *N* = 4 (16%)	OR (95% CI)
Mean age (years)	57.7 ± 8.6	64.3 ± 9.8	0.9 (0.8–1.1)
Mean BMI (preop) (kg/m^2^)	25.4 ± 3.7	26.3 ± 3.7	0.9 (0.7–1.3)
ISL classification (%)	I = 10.5	I = 0.0	
	IIA = 68.4	IIA = 50.0	3.3 (0.3–31.1)
	IIB = 21.1	IIB = 50.0	0.3 (0.0–2.9)
ICG stage (%)	II = 31.6	II = 0.0	
	III = 57.9	III = 25.0	16.5 (1.1–250.2)
	IV = 10.5	IV = 75.0	0.1 (0.0–0.9)
Mean years from lymphedema onset	5.9 ± 4.7	8.3 ± 7.4	0.9 (0.8–1.1)
Infections			
Preoperative (%)	28.6	75.0	0.1 (0.0–1.5)
Postoperative (%)	4.0	50.0	0.1 (0.0–1.7)
Mean number of anastomosis	2.0	1.5	3.7 (0.5–27.9)

*OR* odds ratio, *CI* confidence interval, *ISL* International Society of Lymphology, *ICG* indocyanine green

## Discussion

In the current study, in three quarters of patients, at least one anastomosis was considered patent. Compared with the non-patent group, the patent anastomosis group showed more improvement in QoL, a decrease in arm circumference, and a higher discontinuation rate of compression stockings, indicating a conceivable positive correlation between a patent anastomosis and clinical improvement after LVA.

Only a few studies discussed the long-term patency rate, QoL, and arm circumference after LVA in the upper extremities. Recently, Winters et al. showed at least one patent anastomosis in 66.7% of 12 included patients [28]. Other studies examined the patency in the lower extremity after 6 to 12 months, with a patency rate ranging from 44 to 75% [29, 30, 36]. Mukenge et al. conducted LVA in 5 patients with genital lymphedema resulting in a patency rate of 100% after 12 months [31]. In these studies, similar imaging techniques (ICG lymphography) were used for evaluating the anastomosis showing different patterns of anastomosed veins, indicating that veins with different branching patterns can be used in the LVA procedure. Boccado et al. used the Kleinhand Transport Index after lymphoscintigraphy to evaluate the anastomosis [29]. Similar results were obtained on patency rate in the current study (76%).

Winters et al. described both a significant increase in QoL according to the LYMQoL and decrease in arm volume using the water displacement technique after a follow-up of 12 months [37]. Apart from the arm volume, these results are in line with the current study. Differences in results on arm volume may be explained by the different measuring techniques as this study measured the arm circumference instead of volume. Cornelissen et al. did not find a significant difference either, using the same technique [38]. Moreover, although only a slight decrease was presented in arm circumference, the current study showed a reduction in UEL index (− 2.1) in the patent group, while an increase in arm circumference was seen in the non-patent group (+ 4.2). Therefore, a patent anastomosis could have a positive impact on the arm circumference.

Three quarters of the patients with a patent anastomosis could discontinue the compression stockings. Twenty-five percent of the patients with non-patent anastomosis discontinued the compression stockings. This might seem as a high percentage; however, this concerned only one patient with ISL stage IV. Initially, this patient showed a decrease in arm circumference and symptoms, indicating a surgical benefit. Yet, after 1-year follow-up, the UEL index was increased and the anastomosis was shown to be non-patent.

Comparison between the number of anastomosis in relation to patency illustrated that in all patients with three anastomosis at least one was patent, whereas only 66.7% was patent in patients with only one anastomosis. Furthermore, the results presented an odds ratio of 3.7 between patent anastomosis and the number of anastomosis, suggesting a possible association between a higher number of anastomosis per patient and higher patency rate.

Many studies stated that LVA was less effective in advanced lymphedema and in patients presenting ICG stage IV or more [39–43]. Similarly, the current study illustrated a lower patency rate in ISL stage IIB and in ICG stage IV (OR 0.3 and OR 0.1, respectively). On the contrary, ISL stage IIA (OR 3.3) and ICG stage III (OR 16.5) showed a positive correlation with a patent anastomosis, indicating that early stage lymphedema could result in a higher chance of a patent anastomosis as it was postulated before by other authors [44–46].

Pre- and postoperative infections (both OR 0.1) may be associated with a lower chance of a patent anastomosis.

Due to the fact that patients consider the compression stockings as the greatest factor in decreasing QoL and previous studies showed a large discontinuation rate of compression stockings, this was considered one of the secondary outcomes [10, 32].

Postoperatively, MLD was continued in the same frequency, to a lesser degree, or stopped depending on symptoms and swelling of the arm. Therefore, outcomes achieved are a combination of LVA operation and conservative therapy, by maintenance of the arm after surgery if necessary.

Due to the small sample size, statistical analysis showed no significance for the previous described results. Therefore, the results can only be described exploratively.

### Limitations

Although the study population was small (*N* = 25), this is one of the largest one compared to the previous literature till date. Despite the small study population, still a significant increase in QoL was found and difference in patent and non-patent groups could be seen. The power for this study, however, was achieved for analysis of the QoL.

Moreover, only four patients presented non-patent anastomosis. Although this low number could be considered as a good result after LVA procedure, statistical analysis was limited due to the noticeable difference in number of subjects per group (*N* = 19 in patent versus *N* = 4 in non-patent).

Many patients considered ICG injections uncomfortable, thereby refusing to have a postoperative ICG lymphography. These patients were excluded, and therefore, there may be a selection bias in this study.

## Conclusions

Acceptable patency rate after LVA at 12 months was presented. A positive correlation between a patent anastomosis and clinical improvement in terms of QoL, arm circumference, and discontinuation of compression stockings was suggested. Early stage lymphedema and higher number of anastomoses could be associated with better patency rate. However, in the non-patent group, higher incidence of infections might be associated. None of these differences were found to be significant, but a trend could be seen.

Further research with prospective, larger study population is required to provide higher evidence for the correlation between the patent anastomosis after LVA procedure and clinical improvement.

## Electronic supplementary material

Below is the link to the electronic supplementary material.

Supplementary material 1 (MP4 13121 kb)

## Abbreviations

BCRL: breast cancer-related lymphedema
QoL: quality of life
ISL: International Society of Lymphology
LVA: lymphaticovenous anastomosis
ICG: indocyanine green
MLD: manual lymphatic drainage
UEL: upper extremity lymphedema
DM: diabetes mellitus
OR: odds ratio
CI: confidence interval
